# Physical activity and functional preservation in older adults with hip osteoarthritis: A comparative analysis of age cohorts in the SHARE study

**DOI:** 10.1371/journal.pone.0317578

**Published:** 2025-03-26

**Authors:** Graziella Martins de Vasconcellos de Mello, Fernando Tadeu Trevisan Frajácomo, Ariele B. Haagsma, Dyego L. B. Souza, Vanessa Barbosa de Oliveira, Márcia Olandoski, José Rocha Faria Neto, Javier Jerez-Roig, Cristina P. Baena

**Affiliations:** 1 Pontifícia Universidade Católica do Paraná (PUCPR), Paraná, Curitiba, Brazil; 2 Centro Universitário Estácio, Ribeirão Preto, São Paulo, Brazil; 3 Universidade Federal do Rio Grande do Norte, Rio Grande do Norte, Natal, Brazil; 4 Research Group on Methodology, Methods, Models and Outcomes of Health and Social Sciences (M3O), Faculty of Health Sciences and Welfare. Centre for Health and Social Care Research (CESS), University of Vic-Central University of Catalonia (UVic-UCC), Barcelona, Spain,; 5 Institute for Research and Innovation in Life Sciences and Health in Central Catalonia (IRIS-CC), Vic, Spain; 6 Department of Health Promotion and Rehabilitation, Lithuanian Sports University, Kaunas, Lithuania; University of Naples Federico II: Universita degli Studi di Napoli Federico II, ITALY

## Abstract

**Background:**

Hip osteoarthritis (HOA) is a major contributor to functional impairment in older adults. Physical inactivity and comorbidities are commonly associated with declines in functional ability. However, the relationship between physical inactivity and functional outcomes in individuals with HOA, particularly those aged 80 years and older, remains underexplored.

**Objective:**

This study investigated the association between physical inactivity and functional limitations in older adults with HOA, focusing on two age groups: 60–79 years and 80–100 years.

**Methods:**

We analyzed data from Wave 5 of the Survey of Health, Aging, and Retirement in Europe (SHARE), constructing univariate and multivariable logistic regression models. Functional limitations served as dependent variables, with physical inactivity as the primary explanatory variable and number of chronic conditions, body mass index (BMI), gender, education, and depression as covariates.

**Results:**

The study included 2,088 participants (mean age 73.1 ± 8.5 years; 73.7% female). Physical inactivity was reported by 16.8% (n =  261) of participants aged 60–79 years (n =  1,556; 72.5% female) and 49.6% of those aged 80–100 years (n =  532; 22.5% female). Poor handgrip strength and difficulty rising from a chair were significantly more prevalent among females aged 60–79 years (p <  0.002 for both). A marked decline in both physical activity and functional ability was observed between the two age groups. Physical inactivity emerged as an independent risk factor for reduced functioning across all outcomes, with stronger associations in the 80–100 years age group.

**Conclusions:**

Physical inactivity is a key predictor of functional decline in older adults with HOA, with its impact being particularly pronounced among those aged 80 years and older. These findings highlight the importance of physical activity to preserve functional abilities in this population.

## Introduction

According to data from the European Project on Osteoarthritis (EPOSA), osteoarthritis (OA) is the leading cause of pain and disability among older people [1]. The Global Burden of Disease Study [2] projected that 595 million people (7.6% of the world’s population) lived with OA in 2020, with higher incidence amongst females. In people with OA, movement limitation is standard, with one in every four people over 60 unable to perform activities of daily living such as dressing, getting out of a chair, or walking rapidly [3].

Physical inactivity, obesity, comorbidities and biomechanical variables all represent modifiable risk factors that can affect the progression of pain and physical function in people with OA [4] and current research suggests that higher levels of physical activity (PA) are inversely associated with the prevalence and incidence of comorbidities in people with hip and knee OA [5,6]. Still, the relationship between PA and function remains to be described, particularly among the oldest-old population. A Cochrane review of twenty-one trials found that involvement in exercise programs slightly enhanced physical function in those with OA aged 45 and over [7]. Furthermore, clinical guidelines recommend exercise and PA programs as the primary components for managing and preventing significant OA [8]. However, programs aimed to increase both self-reported and accelerometry-measured PA demonstrated limited effectiveness [9] and the relationship between PA and other risk factors for physical function is still unknown in early older and late older people with OA.

Population-based research is essential for understanding the pattern and link between PA, comorbidities, and physical function. A previous systematic review has described a complex interplay of physical, psychological, and social factors influences PA engagement in individuals with lower limb OA [10]. Still, there is scarce evidence on the factors influencing physical function in older people with hip osteoarthritis (HOA) in an older than 80 years population. Utilizing the European Health, Aging, and Retirement Survey (SHARE), we aimed to analyse the association between PA levels and self-reported daily activities among older adults with symptomatic HOA, segmented by two age groups (60–79 and 80–100).

## Methods

### SHARE database

This is a cross-sectional analysis of SHARE, a global prospective survey that aims to investigate the aging process within different social and cultural backgrounds across Europe. The SHARE Project complies with the Declaration of Helsinki’s guidelines for participant confidentiality; all participants and/or their legal guardians provided informed consent for study participation. SHARE is subject to continuous revisions by ethical committees, waves 1–4 were approved by the Manheim University Ethical Committee, while the subsequent waves were approved by the Ethics Council of the Max Planck Society. Additional information on sampling processes, data collection, and other methodological features can be obtained at www.share-project.org [11].

The SHARE study has gathered socio-demographic characteristics, medical history, functionality, and cognitive function scores of individuals age => 50 in 28 different European countries. All households with at least one person born in 1954 or earlier and speaking the national language were included. More details about SHARE can be found elsewhere [11]. SHARE provides specific training before the evaluators visit the respondents’ homes for each wave. The SHARE survey started in 2004 with its first wave, and was repeated every two years, with new participants, nations, and factors added each time [12].

For this analysis, we included those participants with => 60 years of age that reported hip osteoarthrosis.

### Outcome measurements

Functional limitations were assessed using self-reported mobility and other physical functioning issues. Difficulty getting up from a chair after sitting for long periods was assessed using self-reports of mobility and other physical functioning characteristics using “yes” or “no” questions that potentially predict mobility capacity [13]. Difficulty putting on shoes and socks also indicates HOA [14].

HGS was determined in kilograms using a portable dynamometer (Smedley, dynamometer S, TTM, Tokyo, 100 kg). HGS is known for being able to predict disability, morbidity, frailty, and mortality [15,16] and is utilized as a general health indicator, with levels decreasing with age [17]. Participants were instructed on HGS measurement techniques and allowed to use the dynamometer twice in each hand. They were advised to sit with their arms resting on their torso and their elbows flexed at 90°. The final HGS value was determined by combining the maximum HGS measurements from both hands. The variable was generated exclusively for respondents with two valid measurements for each hand, defined as values ranging from 0 to 100 kg with a difference of no more than 20 kg. The European Working Group on Sarcopenia in Older People 2, 2019 edition [18] establishes cutoff values to diagnose sarcopenia for men and women at < 27 kg and < 16 kg, respectively.

### Exposure measurement

Physical inactivity was originally described as two categorical variables according to the frequency of vigorous and moderate PA (1. more than once a week; 2. once a week; 3. once to three times per month; and 4. virtually never or never). Vigorous activities included sports, heavy housework, and physically demanding employment, while mild activities included gardening, car cleaning, and walking. For this study, physical inactivity was defined as low level of PA or lack of moderate or vigorous activity.

### Covariables measurements

Within the demographic variables, respondents indicated whether and how frequently they drank alcohol (“never,” “1 to 2 times a month,” “1 to 2 times a week,” “3 to 4 times a week,” and “almost every day”). Their smoking status was determined to be either current smokers or nonsmokers. The number of chronic conditions were self-reported, which limited the study’s scope. The chronic conditions included hypertension, high cholesterol, stroke, cerebrovascular disease, diabetes, chronic lung disease, asthma, arthritis, osteoporosis, cancer, duodenal ulcer, Parkinson’s disease, cataracts, and hip and femoral fractures. Multimorbidity was characterized by three or more chronic conditions, which are associated with a lower quality of life and mortality [19,20].

Self-reported weight and height were used to compute BMI (kg/m^2^), which was then classified according to the World Health Organization categories: underweight (<18.5), average (18.5-24.9), overweight (25-29.9), and obese (≥30).

Euro-D, a mental health questionnaire composed of a scale through several symptoms (depression, pessimism, suicidal tendencies, guilt, sleep, interest, irritability, appetite, fatigue, concentration, pleasure, and crying), was used to measure depression symptoms (yes if 4-12 points and no if ≤  3 points).

### Statistical analysis

Statistical analyses were conducted using IBM SPSS Statistics software, version 29.0.2 (IBM Corp., Released 2020, Armonk, NY). All variables were categorical and are presented as absolute frequencies and percentages.

Comparisons between genders were performed using Fisher’s exact test. Logistic regression models were fitted for univariate and multivariable analyses to identify factors associated with the outcomes of interest, limitations in activities of daily living (none or at least one), poor handgrip strength (defined as ≤  26 kg for men or <  16 kg for women), difficulty in dressing including shoes and socks (yes or no), and difficulty getting up from a chair (yes or no).

Physical inactivity was the exposure variable and covariables included gender, years of education (≤8, 9-12, > 12 years), current smoking status (yes or no), alcohol consumption (once or twice a month or at least once a week), physical inactivity (yes or no), depression (yes or no), history of heart attack (no or ever diagnosed/currently having), hypertension (yes or no), high blood cholesterol (yes or no), history of stroke (yes or no), and diabetes or high blood sugar (yes or no).

Initially, each variable was analyzed individually to assess its association with each functionality variable.

Only variables with statistically significant associations in the univariate analysis were included in the final multivariable model to determine the independence of effects.

Significance of each variable in the model was assessed using the Wald test, with the association measure reported as the odds ratio (OR) with a 95% confidence interval (95%CI).

Multicollinearity was examined prior to analysis, and model fit was assessed using the Hosmer-Lemeshow test. A complete case analysis was conducted, as missing data were few and considered random, with no observable pattern or bias. Statistical significance was set at p <  0.05.

## Results

### Study population characteristics

**Fig 1:**
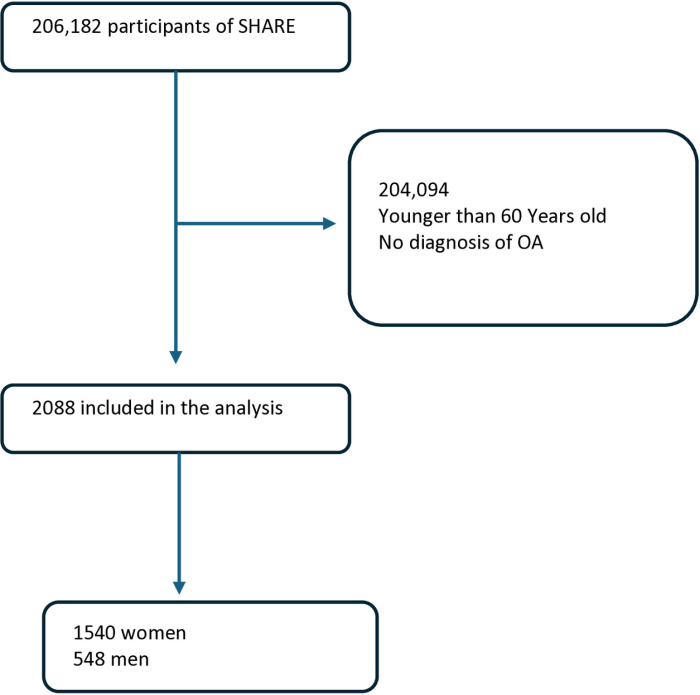
Flowchart of the studied population.

Our analysis included 2,088 participants: 1,540 (73.8%) women and 548 men as shown in Fig 1. The study participants were predominantly nonsmokers (72.8%). The mean age was 72.5 ( ± 8.2) for men and 73.4 ( ± 8.5) for women. The 60-79 subgroup had a mean age of 69.1 ( ± 5.5) for men and 69.3 ( ± 5.5) for women. The mean age between 80 and 100 years was 84.6 ( ± 3.7) for men and 84.6 ( ± 3.9) for women. Among the studied population, 25.1% (n =  525) was physically inactive. The 80-100 group showed the highest rates of physical inactivity (women 49.8% for women and 49.2% for men). Table 1 shows the demographic description of our population. While schooling categories were equally distributed among male and female subgroups in the 60-79 years of age group, females in the 80-100 years of age group majorly reported less than 8 years of education. Man in both age groups reported consuming alcohol more frequently than women. The majority of male and female in both age groups reported 2-4 comorbidities, being high blood pressure and high cholesterol the commonest.

**Table 1:** Participants sociodemographic and clinical characteristics.

Variable	All (n = 2,088)	Age 60-79 y		Age 80-100 y	
		Male (n = 428)	Female (n = 1,128)	Male (n = 120)	Female (n = 412)
Years of schooling					
≤8	778 (37.4%)	125 (29.3%)	393 (34.9%)	41 (34.2%)	219 (53.5%)
9-12	778 (37.4%)	169 (39.6%)	442 (39.3%)	38 (31.7%)	129 (31.5%)
> 12	526 (25.3%)	133 (31.1%)	291 (25.8%)	41 (34.2%)	61 (14.9%)
Smoke at the present time	302 (17.2%)	99 (25.8%)	169 (18.3%)	12 (10.7%)	22 (6.5%)
Alcohol at least once a week	754 (36.1%)	259 (60.5%)	343 (30.4%)	65 (54.2%)	87 (21.1%)
BMI (kg/m^2^)					
<25	608 (30.2%)	92 (21.7%)	315 (29%)	50 (42%)	151 (39.1%)
25-29.9	763 (37.8%)	193 (45.5%)	378 (34.8%)	46 (38.7%)	146 (37.8%)
≥30	645 (32%)	139 (32.8%)	394 (36.2%)	23 (19.3%)	89 (23.1%)
Physical Inactivity	525 (25.1%)	63 (14.7%)	198 (17.6%)	59 (49.2%)	205 (49.8%)
Heart attack: ever diagnosed/currently having	413 (19.8%)	81 (18.9%)	189 (16.8%)	40 (33.3%)	103 (25%)
High blood pressure or hypertension	1178 (56.4%)	238 (55.6%)	627 (55.6%)	70 (58.3%)	243 (59%)
High blood cholesterol	697 (33.4%)	137 (32%)	389 (34.5%)	35 (29.2%)	136 (33%)
Stroke	148 (7.1%)	32 (7.5%)	60 (5.3%)	17 (14.2%)	39 (9.5%)
Diabetes or high blood sugar	409 (19.6%)	84 (19.6%)	222 (19.7%)	30 (25%)	73 (17.7%)
Number of chronic conditions					
1	224 (10.7%)	51 (11.9%)	130 (11.5%)	13 (10.8%)	30 (7.3%)
2 - 4	1210 (58%)	260 (60.7%)	667 (59.1%)	66 (55%)	217 (52.7%)
≥ 5	654 (31.3%)	117 (27.3%)	331 (29.3%)	41 (34.2%)	165 (40%)

### Handgrip strength and PA associations

**Fig 2:**
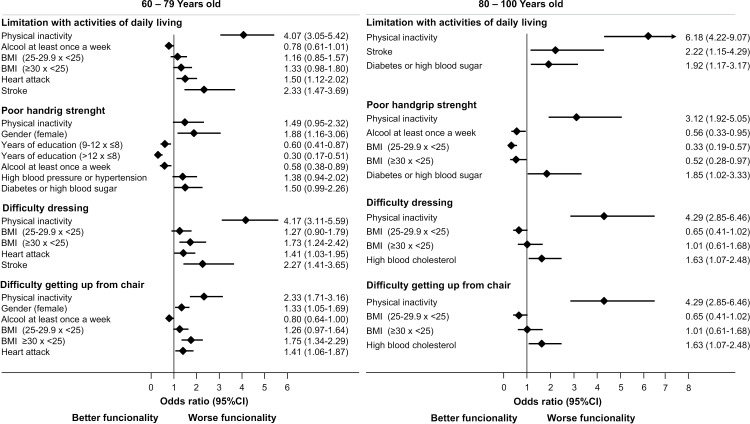
Multivariable model results for the association between functional limitations and physical inactivity with co-variables in age groups.

Mean HGS was 23,8 ± 6,7 and 40,7 ± 9 kgf for female and male among the 60-79 years of age group and 18,6 ± 5,5 and 30,4 ±  7,2 for female and male respectively among the 80-100 years of age group. Low HGS was present in 14.9% of the studied population (n =  264). The 80-100 subgroup presented with the lowest rates of low HGS compared to the 60-79 subgroup (p < 0.001). While 30.5% of women in the older group presented with low HGS, only 12.7% had low HGS in the 60-79 subgroup. The same was observed within the male population (26% vs 5.3%, 80-100 vs 60-79) (Table 2). Multivariable models shown in Fig 2 revealed that being female was associated with decreased HGS among participants aged 60 to 79. Having more than eight years of schooling and alcohol consumption at least once a week were protective factors for HGS in the 60-79-year-old subgroup.

**Table 2:** Functionality measures of the studied population by sex and age group.

Variable	All	Age 60-79 y		*p* ^*^	Age 80-100 y		*p* ^*^
		Male	Female		Male	Female	
At least one limitation with activities of daily living	724/2087 (34.7%)	122/428 (28.5%)	324/1127 (28.7%)	0.950	57/120 (47.5%)	221/412 (53.6%)	0.254
Poor handgrip strength (<16 female; < 27 male)	264/1769 (14.9%)	23/393 (5.9%)	124/978 (12.7%)	**<0.001**	25/96 (26%)	92/302 (30.5%)	0.442
Difficulties in dressing, including shoes and socks	526/2087 (25.2%)	100/428 (23.4%)	244/1127 (21.7%)	0.494	42/120 (35%)	140/412 (34%)	0.828
Difficulties in getting up from chair	1225/2088 (58.7%)	209/428 (48.8%)	652/1128 (57.8%)	**0.002**	77/120 (64.2%)	287/412 (69.7%)	0.266

*Fisher’s exact test, p < 0.05.

Physical inactivity was an independent and significant predictor of reduced grip strength in the 80–100 years of age group, as shown in Fig 2. Also, being overweight was a significant protective factor for the 80-100 years of age group.

### Limitations in everyday activities and PA associations

Limitations in everyday activities were observed in 34.7% of individuals (n =  724) in the overall population. Compared to the 60-79 age group, the 80-100 age group experienced a higher rate of limitation with activities of daily living. Multivariable models identified physical inactivity and stroke as significant predictors of difficulties in activities of daily living in both the 60-79 and 80-100 age groups. Physical inactivity was also associated with a fourfold chance of difficulty in getting dressed in people over 60 with HOA. Regarding comorbidities diabetes was a significant predictor of restrictions in activities of daily living in the 80–100 years of age group.

Dressing difficulties, including putting on shoes and socks, was reported in 25.2% (n = 526) of the overall population (Table 2). Among the of 60-79 years old group, 21.7% (n = 244) of women and 23.4% (n = 100) of men were unable to perform such task, while among the group 80-100 years of age women (34%; n = 140) and men (35%;n = 42) reported it similarly. According to multivariable analysis, physical inactivity was a significant and independent variable associated with difficulties dressing in both age groups.

Stroke, heart attack, and obesity were associated with difficulties with dressing in those in the 60 to 79 years of age group. Meanwhile, high blood cholesterol was associated with difficulty getting dressed among individuals aged 80–99.

It was also observed that 58.7% (n = 1225) of the population reported difficulty getting up from a chair after long periods of sitting. In the 60-79 age group, 57.8% (n = 652) of women and 48.8% (n = 209) of men failed to complete the task, whereas in the 80-100 age group, 69.7% (n = 287) of women and 64.2% (n = 77) of men struggled with such daily activity.

Multivariable models confirmed that physical inactivity was an independent exposure for difficulties getting up from a chair in both age groups (Fig 2). In addition, past heart attacks and obesity were also associated with difficulties getting up from a chair in the 60–79 years of age group. In the 80-99 years of age group, high blood cholesterol was significantly associated with difficulty getting up from a chair.

## Discussion

This population-based study examined the independent associations between physical inactivity, gender, BMI, years of schooling, alcohol intake, and specific comorbidities and functionality in two age subgroups (60-79 and 80-99 years old) of people with HOA.

We found in both age groups that self-reported physical inactivity was an independent predictor of low muscle strength measured by handgrip and functional decline defined by self-reported limitations in daily activities. Morbidities such as stroke, heart attack, and obesity were associated with poor functionality in people aged 60 to 79. In contrast, stroke, diabetes, and high blood cholesterol were major factors for decreased functionality in the 80–100-year-old subgroup.

Our findings highlight that physical inactivity is the primary predictor of impaired functionality in older people with HOA. These results are consistent with previous research. A prospective community-based study using self-reported PA in people with hip or knee OA (mean age 65.4 years) found that regular or very regular PA was linked to long-term improvements in physical function [21]. A systematic and comprehensive review found compelling evidence that PA reduces pain and enhances physical function in adults with hip or knee OA [6]. Despite the known association between PA and functioning, we observed that advanced age (over 80 years old) favours the importance of PA as a key component of physical function in older people with HOA. Given the magnitude and the independence of physical inactivity in both groups, it is possible to hypothesize that the mere fact of not being inactive could lower this impact.

Current OA guidelines recommend using land-based exercise as the first line of treatment for hip and knee OA [8,22]. In the literature on OA, the terms “PA” and “exercise” interchangeably refer to various aspects of human movement. PA is defined as any energy expenditure from skeletal muscle above resting levels, which helps to prevent and manage OA. Meanwhile, planned, structured, and repeatable PA is known as exercise [23]. Our population study only considered self-reported PA levels and discovered an independent link between low PA levels and reduced functioning. These findings highlights the current OA guidelines’ recommendation to enhance PA levels to maintain physical function even though anecdotal evidence shows that some older patients with OA are recommended to make rest.

The maintenance of physical function through PA happens through mechanisms that enhance joint stability while reducing pain and inflammation. Stair patterns, particularly stair ascent, vary among people with HOA. Yet, focused therapy may ease discomfort and improve mobility by transferring load and relieving pressure on hip abductors [24]. Furthermore, regular PA is necessary for adjusting muscle and neuromuscular function to compensate for joint deterioration. Research reveals that neuromuscular training or classical quadriceps strength can reduce muscle discomfort and enhance muscle function without affecting knee alignments in hip or knee OA [25,26].

In addition to biomechanical benefits, PA promotes joint function by enhancing overall physical conditioning and lowering inflammation. Preoperative PA helps to maintain muscle stiffness and function following joint replacement operations, such as total hip arthroplasty [27]. Regular exercise also has anti-inflammatory properties because it helps to break the vicious cycle of local synovial membrane inflammation and systemic inflammation caused by numerous morbidities [28], which can, in turn help reduce OA-related inflammation and discomfort. These physiological advantages underline the need for maintaining an active lifestyle to control OA symptoms, underscoring exercise’s dual role in both mechanical and biochemical disease progression management. Overall, these systems work to alleviate symptoms and improve the quality of life for those with OA.

This study has some limitations. The cross-sectional design of our analysis prevented us from investigating a causal association between physical inactivity and functional outcomes. Also, SHARE is a survey; hence, there were no imaging tests to assess the severity of HOA. Another limitation is the lack of objective measures of PA, such as pedometers and accelerometers. The survey revealed that 25.1% of people in the studied population were physically inactive, indicating a group with high self-reported activity levels. This variation is significant, especially compared to other SHARE research and an age-matched group [29]. One possible explanation is that self-reported PA questionnaires may overestimate data [30].

A strength of this study was the sample size, which represented an exponentially rising but understudied population. Moreover, our study included various functional variables for both sexes and determinants of independence and quality of life, such as strength, mobility, and endurance. Furthermore, after accounting for frequent confounders, our robust models demonstrated the impact of physical inactivity and multimorbidity on functioning. To our knowledge, this is the first study to compare functional measurements and their relationships across these two age groups.

Furthermore, the study findings could imply that sustaining physical exercise is critical even in individuals older than 80 years old. However, the amount of PA in those specific age groups that would increase their levels of functionality remains to be measured by proper interventions. These findings reinforce to health care practitioners, that implementing and maintaining PA-based programs is the foundation of physical functional preservation in older people with HOA.
